# Genotyping analysis of *Helicobacter pylori *using multiple-locus variable-number tandem-repeats analysis in five regions of China and Japan

**DOI:** 10.1186/1471-2180-11-197

**Published:** 2011-09-03

**Authors:** Chunliang Guo, Yaling Liao, Yan Li, Jun Duan, Ying Guo, Yuqian Wu, Yujun Cui, Hongwu Sun, Jinyong Zhang, Bing Chen, Quanming Zou, Gang Guo

**Affiliations:** 1 Department of Clinical Microbiology and Immunology, College of Medical Laboratory, Third Military Medical University, Chongqing, 400038, China; 2 Department of Endocrinology, Southwest Hospital, Third Military Medical University, Chongqing, 400038, China; 3 Department of clinical laboratory, 155 Central Hospital of PLA, Kaifeng, 475000, China; 4 Medical Research Center, Southwest Hospital, Third Military Medical University, Chongqing, 400038, China; 5 Institute of Agriculture and Life Science, Chongqing University, Chongqing 400030, China; 6 Laboratory of Analytical Microbiology, State Key Laboratory of Pathogen and Biosecurity, Institute of Microbiology and Epidemiology, Beijing, China; 7 National Engineer Research Center for Immunization Products, Third Military Medical University, Chongqing, 400038, China

## Abstract

**Background:**

*H. pylori *(*Helicobacter pylori*) is the major causative agent of chronic active gastritis. The population of *H. pylori *shows a high genomic variability among isolates. And the polymorphism of repeat-units of genomics had participated the important process of evolution. Its long term colonization of the stomach caused different clinical outcomes, which may relate to the high degree of genetic variation of *H. pylori*. A variety of molecular typing tools have been developed to access genetic relatedness in *H. pylori *isolates. However, there is still no standard genotyping system of this bacterium. The MLVA (Multi-locus of variable number of tandem repeat analysis) method is useful for performing phylogenetic analysis and is widely used in bacteria genotyping; however, there's little application in *H. pylori *analysis. This article is the first application of the MLVA method to investigate *H. pylori *from different districts and ethnic groups of China.

**Results:**

MLVA of 12 VNTR loci with high discrimination power based on 30 candidates were performed on a collection of 202 strains of *H. pylori *which originated from five regions of China and Japan. Phylogenetic tree was constructed using MLVA profiles. 12 VNTR loci presented with high various polymorphisms, and the results demonstrated very close relationships between genotypes and ethnic groups.

**Conclusions:**

This study used MLVA methodology providing a new perspective on the ethnic groups and distribution characteristics of *H. pylori*.

## Background

*Helicobacter pylori *(*H. pylori*) is a spiral-shaped, Gram-negative bacterium that infects half the world's population and is the major cause of chronic gastritis, peptic ulcers and gastric malignancies, including gastric non-cardia adenocarcinoma and mucosal-associated lymphoid tissue lymphoma [1,2]. Most infected individuals present with no clinical symptoms, but approximately 10~20% will develop peptic ulcers and 1% will develop gastric cancer [3,4], which could be associated with the diversity of *H. pylori*.

*H. pylori *exhibits exceptionally high rates of DNA point mutations and intra- and inter-genomic recombination. Recently, many molecular typing tools have been developed to investigate genetic relatedness among *H. pylori *isolates. However, these methods have limitations including lower discrimination power, or preventing results from different labs being compared [5,6].

In 1999, MLVA analysis was proposed as a general approach to providing accurate, portable data that were appropriate for the epidemiological investigation of bacterial pathogens [7-11]. However, there's little information concerning populations of *H. pylori *species using MLVA. Whether this method is available for the *H. pylori *population is still uncertain.

*H. pylori *infections in China are common and extensively distributed, with an average infection rate of about 58%. In this study, 12 VNTR loci of the *H. pylori *genome were identified and used to analyze 202 strains of *H. pylori *which originated from different regions of China and Japan.

## Results

### Multi-VNTR loci for *H. pylori *genome

PCR products amplified from the reference strains 26695, HPAG1 and J99 were identical to the published sequences sizes. Of the locus VNTR-2576 and VNTR-614, the PCR products sequencing were consistence with our electrophoresis results. The exact number of tandem repeats at each locus could be determined from the sizes of the PCR products.

In this study, 30 VNTR loci were candidated from the *H. pylori *database. And we finally identified 12 VNTR loci using analysis, which were also distributed throughout the *H. pylori *genome (Table 1). There's no variation in the other 18 loci, which were removed in the following study. The variation in repeat numbers is divergence at the 12 VNTR loci. The main characteristics of the 12 VNTR loci are listed in Table 2, including the diversity index of each locus.

**Table 1:** Characteristics of the 12 VNTR loci in the reference *H.pylori *strains

Locus name	Position in the reference strains (bp)			Number of repeat times			Repeat unit size (bp)	Related gene in 26695
	26695	HPAG1	J99	26695	HPAG1	J 99		
VNTR-180	16605. . 16643	17912. . 17932	16761. . 16778	2	1	1	20	-
VNTR-263	42061. . 42115	43125. . 43167	42199. . 42252	4	3	4	14	rfbD
VNTR-614	129983. . 130389	125875. . 126119	1238315. . 1238474	9	5	3	53	dld
VNTR-557	120659. . 120675	118007. . 118023	116640. . 116673	1	1	2	17	-
VNTR-606	129957. . 130396	1189474. . 1189690	1238289. . 1238481	3	1	1	138	dld
VNTR-1801	485276. . 485316	452649. . 452673	448197. . 448261	1	1	2	27	hsdR
VNTR-2181	580530. . 580546	546643. . 546659	544199. . 544227	1	1	2	12	-
VNTR-2457	665196. . 665241	628875. . 628996	625968. . 626121	1	3	3	54	ppa
VNTR-2576	696789. . 697001	1067559. . 1067708	1112077. . 1112164	10	7	4	21	galU
VNTR-5062	1382502. . 1382594	1314612. . 1314776	1360215. . 1360348	8	14	11	12	-
VNTR-5282	1439274. . 1439284	1368268. . 1368279	1412390. . 1412413	1	1	2	12	clpX
VNTR-5581	1512724. . 1512751	1419518. . 1419531	1464638. . 1464651	2	1	1	14	-

**Table 2:** Description of 12 VNTR loci analyzing with 202 *H.pylori *clinical isolates

Locus	Forward and Reverse primer (F/R)	Annealing temperature (°C)	Expected product length in 26695 (bp)	Product size range	Allele size range(unites)	Total number of alleles	Nei's diversity index
VNTR-180	F:TAAAGTGAAAGCGTTACAAAAAGAC R:CTTCAGGGTAGGAATACAGCAGAGT	53	185	165-225	1-4	4	55. 7
VNTR-263	F:TTGAATTGCAAGCTAATGAGTC R:AGAAGTGTTGATGCTAGAAGAG	52	352	310-366	1-5	5	63. 0
VNTR-614	F:ATTGATTATGATTTTCTTGGCAATTTTG R:GCTTATGAATGTGTGTTTTGCTGATGAC	54	758	334-864	1-7, 11	9	80. 7
VNTR-557	F:ATGGAAGTTTTTGATTTGATTG R:GGTGTAATGGGTGTTGATGGTC	50	152	152-202	1-3,	3	12. 3
VNTR-607	F:GAATTGATTATGATTTTCTTGGCAAT R: GCTGAAAACGCTAGGGATAGAGC	52	668	233-673	1, 2, 5-21, 23	20	92. 8
VNTR-1801	F:GCCGTATTTTAGGATAAAGCAAAG R:CGCGTTTTATAGCGCTTCTTATT	52	280	280-604	1-5, 12	5	57. 3
VNTR-2181	F:TTATGGAAAATATCATACAACCCCCTAT R:ATTTAGAAAAATTACCCCTTTCATCAAG	52	378	378-426	1-3, 5	4	20. 9
VNTR-2457	F:TAGAAGATTGCTTGAAAAGCCCTTT R:GCTCTATGATTTTAAAACGCTCCGT	52	650	650-812	1-4	4	73. 6
VNTR-2576	F:GATTTTTGATARGCTTTGCGATAG R:TAAAACGATTTTAGAAAACGACAC	51	371	182-371	1-7, 10	8	46. 2
VNTR-5062	F:AAGCTCGCCCTCATCGCC R:TAAAAAATATTAAATAATCAATT	50	307	223-259	1-4	4	40. 9
VNTR-5282	F:CCTTAAGCTCTTTAGGGGCTGG R:GAGAGTTCTAGGGGCGTGGC	56	335	335-371	1-4	4	36. 2
VNTR-5581	F:CGTTCACTCTGAGCCAGGATC R:GCTCTTTCTGTTTTGTTGTTGTAAT	52	202	190-218	1-3	3	34. 3

### Clustering trend of the strains from different regions and ethnic groups

**Figure 1:**
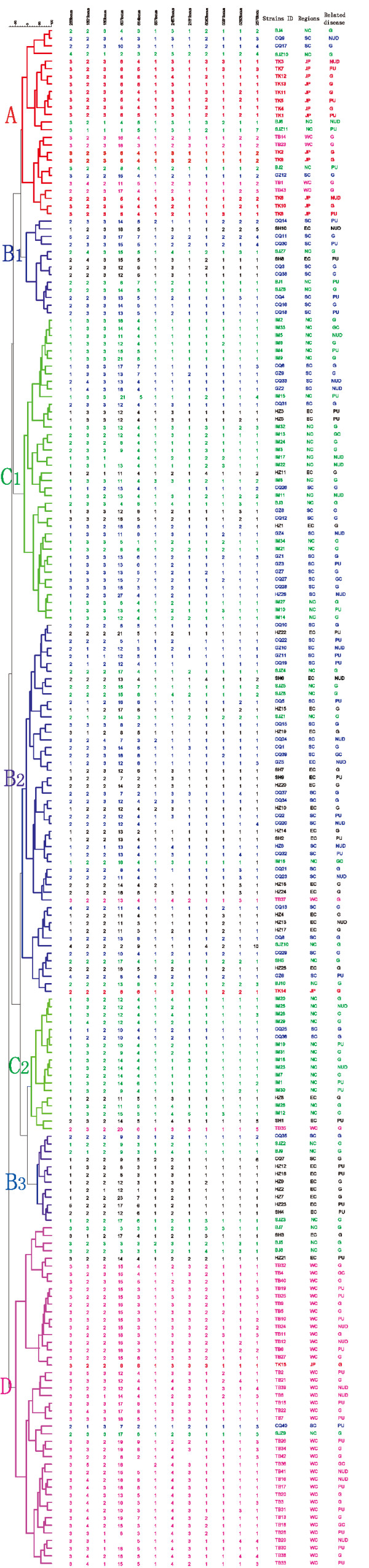
**Dendrogram analysis based on 12 VNTR loci for the 202 *H. pylori *isolates**. Clustering analysis of Neighbor-joining tree (N-J) was using the categorical distance coefficient and the wards method. From left to right, the columns designated to the 12 VNTR loci, the strain ID, geographic origin (location) and *H. pylori *related disease. NC, SC, EC and WC under the column of 'Region' stand for the Southern, Northern, Eastern and Western of China respectively. Disease NUD and G represents the non-ulcer dyspepsia (NUD) and gastritis. And diseases PU (peptic ulcer) comprise duodenal and gastric ulcer as well as disease GC is with the gastric cancer. The branches color code reflects the focus of origin, the same color of the columns stand for origin from the same geographic origin (location). Isolates from different regions showed a certain cluster tendency, as Tokyo isolates were clustered into Group A, Southern and Eastern China isolates were clustered into group B, Northern China were clustered into two major branches, groups C1 and C2. Western China isolates were clustered into group D. While there's no significant relationship between MTs and *H. pylori *related diseases.

A MLVA system to the molecular typing of *H. pylori *strains has been developed. On the basis of the 12 VNTR loci, the profiles of each isolate were obtained (Figure 1). The clinical *H. pylori *strains were divided into 127 MTs, which has not been described previously. According to cluster analysis, most strains from the same focus presented with the same or similar MTs (Figure 1). In addition, strains from the same focus were dispersed in the cluster tree. As shown in Figure 1, the 86.7% (13/15) of the Tokyo isolates had very similar MTs and could be clustered into Group A. One of the remaining Tokyo isolates belonged to the Group C, and the others were scattered distribution. Of the Southern and Eastern Chinese isolates, 74.4% (43/32) were clustered into group B, including B_1_, B_2 _and B_3 _subgroups, and the rest strains were related to Group A, C and D. Of the isolates from Northern China, 60.7% were clustered into two major branches, groups C_1 _(37.5%, 21/56) and C_2 _(23.2%, 13/56), and other strains were scattered. Of the Western China isolates, 86.0% (37/43) were clustered into group D. The strains Tibet 1, 14, 23 and 43 were related to Group A, Tibet 37 and Tibet 35 were related to Group B_2 _and C_2_.

**Figure 2:**
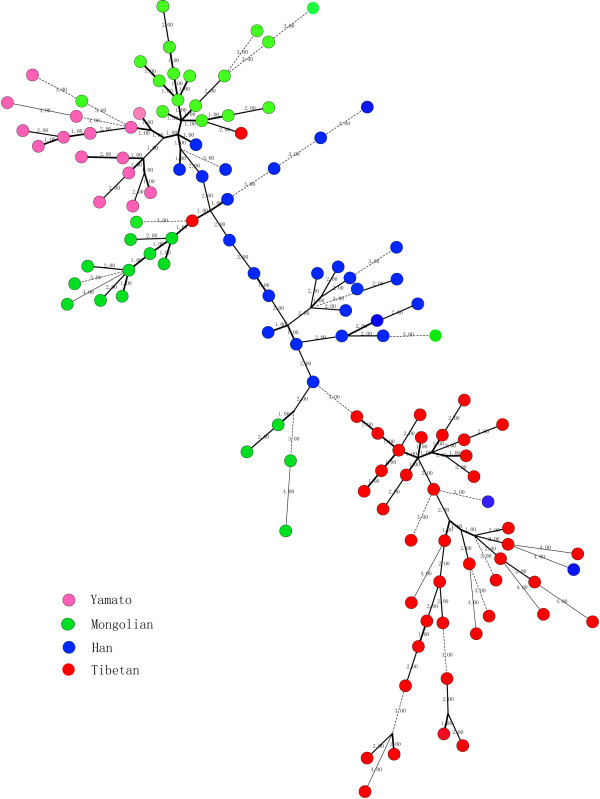
**Minimum spanning tree analysis**. Minimum spanning tree analysis of MTs on the 118 strains from four ethnic groups was constructed. There're 3 kinds of lines, solid, thin and dotted, which represented single, double and triple or more loci variation respectively. The circles stand for strains, and different colors represent different ethnic groups. Strains from different ethic group could group together, esp. of strains from Tibetan.

A minimum spanning tree was constructed on the basis of strains from different ethnic groups: 43 Tibetan, 33 Mongolian, 15 Yamato as well as 27 Han (Figure 2). There was a tendency to cluster into four main subgroups. However, there're still some exceptions, such as the Hangzhou-12 and 21, of Han strains (associated with gastritis and peptic ulcer), were related to the Tibetan strains group. Tibetan strains 1 and 43 (gastritis), were related to the Mongolian group, and Mongolian 16, (gastric cancer), was related to the Japanese group.

### Correlation between *H. pylori *MTs and the related diseases

Among the 202 samples, 14.9%, 55.9%, 25.2% and 4.0% of patients presented with non-ulcer dyspepsia (NUD), gastritis (G), peptic ulcer (PU) and gastric cancer (GC), respectively. And in our study there's no significant relationship between the *H. pylori *MTs and the related diseases.

## Discussion

Recently, many bacterial genomes have been fully sequenced, and analysis of the sequenced genomes has revealed the presence of variable proportions of repeats, including tandem repeats. Short repeat motifs undergo frequent variation in the number of repeated units. MLVA is an appropriate method for bacterial typing and identification, for determining genetic diversity, and for the tracing-back of highly mono-morphological species [12-14]. The MLVA typing was reported to have a high-quality species identification capability and a high discriminatory power. The method has been used in the analysis of many bacteria [15-18], but little research has been carried out in *H. pylori*. Therefore, this study established the *H. pylori *MLVA system and applied to type clinical strains.

The *H. pylori *genome has a number of repeat sequences, and their repeat number results in divergence. The 12 loci identified were distributed throughout the genome. These loci had different variations in different isolates and were able to typing *H. pylori *successfully.

The *H. pylori *MTs were clustered with ethnic groups, consistent with the previous reports [19,20]. The Han strains were selected from Southern China and had little relationship to Mongolian strains from Northern China or Tibetan strains from Western China. It may demonstrate an apparent cluster tendency in different regions and ethnic groups, but there were some exceptions, which may because, unlike other Asian countries with relatively homogeneous populations, China has a heterogeneous population from various ethnic groups. Therefore, there may be more opportunity for DNA transfer between strains of different genotypes in China than other countries. While Tibet is a relatively closed region, *H. pylori *strains from this area have a good cluster.

The *H. pylori *genome shows a high degree of genetic diversity among strains [21,22], but weakly clonal groupings of different diseases were detected, and these could be superimposed on a pattern of free recombination. And the relationship between particular *H. pylori *genotype and related disease has not been sure.

MLVA is a useful molecular tool for epidemiological investigations and recognition of laboratory cross-contamination [23-25]. VNTR analysis thus provides multiple independent characteristics for phylogenetic analysis. Studies have indicated that MLVA is sufficient to resolve closely related isolates. In contrast, combining loci with lower variability values is suitable for establishing clear phylogenetic patterns among strains that have evolved over a longer time period. Theoretically, the greater the number of loci used, the higher the discriminatory power that can be achieved, and subtler phylogenetic relationships among bacterial strains can be established.

At the present time, the MLVA was established and applied to examine the clonal relationships between *H. pylori *isolates from China and Japan. The loci used in this study provided high discriminatory power and successfully separated isolates of different strains from different geographical areas. And there was a particularly evident of *H. pylori *from Tibet, a relatively closed region, which showed better cluster than other ethnic groups. The data will aid in the development of a genomic polymorphism database of *H. pylori*. We have established a preliminary profile of MLVA but more information is required for a comprehensive profile.

China is a large country containing 56 ethnic groups and a large population. Therefore, further studies are required including isolates from more regions and over several more time-frames.

## Conclusions

The studies indicated that MLVA method, based on 12 VNTR loci, is sufficient to resolve closely related isolates for the purpose of *H. pylori *genotyping analysis. This study used MLVA methodology provided a new perspective on the ethnic groups distribution characteristics of *H. pylori*.

## Methods

### *H. pylori *strains and DNA preparation

A total of 202 *H. pylori *strains were included in this study and the background information of the strains is listed in Table 3. The 187 clinical strains were isolated from various regions of China during 1998 and 2010; an additional 15 strains were presented as a gift by Institute of Medical Science University of Tokyo Japan in 2008. Patients ranged from 12 to 75 years old (mean age 44 years). All the patients reporting the symptoms of gastritis (G), peptic ulcer (PU) or gastric cancer (GC) underwent upper gastroendoscopy for both visual examination and biopsy collection. The strains were isolated from gastric biopsy gastrointestinal endoscopy of selected patients, who had not received non-steroidal anti-inflammatory drugs, proton pump inhibitors or other antibiotics during the last 2 months, revealed that out of 202 patients, 172 had either G, DU or GC and 30 had non-ulcer dyspepsia (NUD). Written consent was taken from all the patients before collection of the biopsy. The study was approved by the ethics review board at Third Military Medical University, and informed consent was obtained from all patients before participation.

**Table 3:** Background information of the 202 *H. pylori *clinical strains

City	Region	Ethnic group	Isolated year	No. of isolates
Chongqing	Southern China	Han	2004-2009	40
Guangzhou	Southern China	Han	2004-2010	12
Inner Mongolia	Northern China	Mongolian	2008	34
Shijiazhuang	Northern China	Han	2002-2003	12
Beijing	Northern China	Han	2004-2009	10
Hangzhou	Eastern China	Han	1998-2004	26
Shanghai	Eastern China	Han	2001-2007	10
Tibet	Western China	Tibetan	2008-2009	43
Tokyo	Japan	Yamato	2007	15

Bacteria were separated and cultured in Skirrow medium with 5% fresh sheep blood at 37°C for 24 h~72 h in a micro-aerobic environment. *H. pylori *genomes were extracted using genomic DNA isolation kits (Omega Biotek Inc). Culture and identification of H. pylori were done by appropriate biochemical tests and amplification of 16S rDNA using species-specific primers

### Selection and identification the VNTR loci of *H. pylori*

VNTR loci were selected from the MLVA database http://minisatellites.u-psud.fr/ASPSamp/base_ms/bact.php by estimating the size of PCR products on agarose gels. The repeat sequence of loci ≥ 10 bp, consistency of repeat unit ≥ 90% and a minimum of two alleles in three reference strains of *H. pylori *(26695, HPAG1, J99) were selected for this research. The locations, copy numbers, sizes of the loci and the gene(s) involved are also listed in Table 1.

### PCR amplification

A PCR reaction mixture (30 ml) containing 10 ng of DNA template, 0.5 mM of each primer, 1 unit of Taq DNA polymerase, 200 mM of dNTPs and 10 × PCR buffer (500 mM KCl, 100 mM TrisHCl (pH 8.3) 25 mM MgCl_2_) was utilized. Amplification was carried out in a DNA thermocycler (MJ Research PTC-225) with denaturation at 94°C for 8 min, followed by 30 cycles of denaturation at 94°C for 45 s, annealing at 52°C for 45 s and elongation at 72°C for 1 min [26]. A 10-min elongation at 72°C was performed after the last cycle to ensure complete extension of the amplicons.

Five μl of the PCR products were run on standard 3% agarose gels in 0.56TBE buffer at 8-10 V/cm. Gel lengths of 10 to 40 cm were used according to PCR product size and repeat unit size. Strains in which alleles had been precisely measured by re-sequencing or by direct comparison with a sequenced reference strain were used (In this study DNA from 26695, HPAG1 and J99 were used for this purpose). Multiple interspersed negative controls containing no DNA were included each time PCR was performed.

PCR products of 202 strains on VNTR-2576 and VNTR-614 sites were sequenced directly with a Taq Dye Deoxy Terminator Cycle Sequencing Kit on an ABI 377 sequencer (Applied Biosystems).

### Data analysis

The number of repeat units in 12 VNTR loci were analyzed and inputted into BioNumerics version 5.1 software (Applied-Maths, Sint-Martens-Latem, Belgium), and gel images were obtained using the BioNumerics software package version 6.0 (Applied-Maths, Sint-Martens-Latem, Belgium) or using UVB gel image analysis. The number of repeat units in each locus was deduced by the amplicon size, flanking sequence length and repeat unit size. Data from agarose gel electrophoresis and UVB gel image analysis, obtained by capillary electrophoresis machines, were imported into BioNumerics by creating a virtual gel image. Gel image data were converted into characteristics data sets. Cluster analysis of Neighbor-joining tree (N-J) was carried out using the categorical similarity coefficient and the Ward method. A minimum spanning tree was inferred using characteristic data from cluster analysis. The polymorphism of each locus was represented by Nei's diversity index [27], calculated as DI = 1-∑(allelic frequency)^2^.

### Reproducibility and stability of 12 VNTR loci via *in-vitro *passage

Twenty clinical strain genomes from China and Japan were amplified and multiple DNA samples from each strain yielded PCR products with identical sizes at all loci. Chongqing26 and Tibet36 each yielded no product at one locus, possibly because of mutations or poor quality DNA samples.

The stabilities of the VNTR loci were investigated in a long-term experiment in which the 20 test *H. pylori *isolates used were sub-cultured into fresh Skirrow medium 30 times by serial passages at two or three day intervals. The DNA from the strains cultivated in each passage was extracted and subjected to MLVA analysis. The results of the VNTR analysis demonstrated no difference in tandem repeat numbers (data not shown).

## List of abbreviations

VNTR: variable-number tandem repeat; MLVA: multiple-locus VNTR analysis; MT: MLVA type; G: gastritis; NUD: non-ulcer dyspepsia; PU: peptic ulcer; GC: gastric cancer; SC: Southern China; WC: Western China; NC: Northern China; EC: Eastern China; CQ: Chongqing; GZ: Guangzhou; IM: Inner Mongolia; SJZ: Shijiazhuang; BJ: Beijing; HZ: Hangzhou; SH: Shanghai; TB: Tibet; TK: Tokyo.

## Authors' contributions

YG, JZ and HS participated in the sequence alignment and drafted the manuscript. YC participated in the sequence alignment. JD, YL and YW participated in the design of the study and performed the statistical analysis. GG, QZ, CG, BC and YL conceived of the study, and participated in its design and coordination and helped to draft the manuscript. All authors read and approved the final manuscript.
